# Beneficial Effects of Resveratrol in Mouse Gastrocnemius: A Hint to Muscle Phenotype and Proteolysis

**DOI:** 10.3390/cells10092436

**Published:** 2021-09-15

**Authors:** Laura Mañas-García, Charlotte Denhard, Javier Mateu, Xavier Duran, Joaquim Gea, Esther Barreiro

**Affiliations:** 1 Muscle Wasting and Cachexia in Chronic Respiratory Diseases and Lung Cancer Research Group, Pulmonology Department, IMIM—Hospital del Mar, Parc de Salut Mar, 08003 Barcelona, Spain; lauramgarcia9@gmail.com (L.M.-G.); c.denhard1@gmail.com (C.D.); jgea@psmar.cat (J.G.); 2 Health and Experimental Sciences Department (CEXS), Universitat Pompeu Fabra (UPF), Barcelona Biomedical Research Park (PRBB), 08003 Barcelona, Spain; 3 Centro de Investigación en Red de Enfermedades Respiratorias (CIBERES), Instituto de Salud Carlos III (ISCIII), 08003 Barcelona, Spain; 4 Department of Pharmacy, Hospital del Mar, Parc de Salut Mar, 08003 Barcelona, Spain; fmateu@parcdesalutmar.cat; 5 Scientific and Technical Department, Hospital del Mar-IMIM, 08003 Barcelona, Spain; xduran@imim.es

**Keywords:** disuse muscle atrophy, chronic conditions, limb muscles, resveratrol, proteolysis, apoptosis, muscle fiber cross-sectional areas, atrophy signaling pathways

## Abstract

We hypothesized that the phenolic compound resveratrol mitigates muscle protein degradation and loss and improves muscle fiber cross-sectional area (CSA) in gastrocnemius of mice exposed to unloading (7dI). In gastrocnemius of mice (female C57BL/6J, 10 weeks) exposed to a seven-day period of hindlimb immobilization with/without resveratrol treatment, markers of muscle proteolysis (tyrosine release, systemic troponin-I), atrophy signaling pathways, and muscle phenotypic features and function were analyzed. In gastrocnemius of unloaded mice treated with resveratrol, body and muscle weight and function were attenuated, whereas muscle proteolysis (tyrosine release), proteolytic and apoptotic markers, atrophy signaling pathways, and myofiber CSA significantly improved. Resveratrol treatment of mice exposed to a seven-day period of unloading prevented body and muscle weight and limb strength loss, while an improvement in muscle proteolysis, proteolytic markers, atrophy signaling pathways, apoptosis, and muscle fiber CSA was observed in the gastrocnemius muscle. These findings may have potential therapeutic implications in the management of disuse muscle atrophy in clinical settings.

## 1. Introduction

Disuse muscle atrophy is relevant in chronic respiratory, cardiac, and kidney diseases, especially in advanced stages. Furthermore, conditions such as prolonged immobilization due to bed rest, critical illness, and bone fractures, among others, also lead to disuse muscle atrophy in patients [1,2,3]. Sarcopenia associated with aging and/or chronic disease further aggravates disuse muscle atrophy in patients. The loss of muscle function and mass has been shown to reduce the survival of the patients independent of other factors or the status of the underlying disease [4,5,6,7]. Additionally, the quality of life of patients is also impaired as a result of muscle atrophy induced by prolonged immobilization [4,5,6,7,8].

Several biological mechanisms and pathophysiological alterations take place in the skeletal muscles of patients following disuse muscle atrophy. Those biological events are involved in the process of muscle protein loss, apoptosis, and autophagy. In animal models of disuse muscle atrophy, events such as increased oxidative stress, proteolysis, autophagy, apoptosis, epigenetic modifications, and metabolic derangements have been demonstrated to take place in the muscles exposed to periods of disuse (e.g., immobilization) [9,10,11,12,13,14]. Moreover, structural alterations including a reduction in muscle cross-sectional area (CSA) and a slow-to-fast fiber type switch have also been reported in animal models of disuse muscle atrophy [15,16,17,18]. The kinetics of key biological processes leading to muscle mass loss in response to disuse were shown in previous investigations in which different groups of mice were exposed to a variety of time periods of hindlimb immobilization [15,16,17,18]. In the same mouse models, the biological events and structural alterations of muscle reloading following splint removal were also studied [15,16,17,18].

Signaling pathways including acetylation status of the transcription factors fork-head box O (FoxO)1 and FoxO3 mediated to a great extent the increase in muscle protein loss in the model of unloading and reloading [15,17]. We concluded from those findings that histone deacetylation through sirtuin-1 activity of the transcription factors FoxO1 and FoxO3 partly mediated the beneficial effects of the gastrocnemius muscle during reloading [15,17]. 

Resveratrol is a natural polyphenol extracted from several plants, such as grapes, peanuts, and red wine, with many beneficial effects on several organs, including muscles. Its high antioxidant potential, antiapoptotic, and antiaging properties have conferred this compound a great popularity [19]. It has also been commonly used as a nutraceutical in both human and animal models [20]. In several species, resveratrol also increased lifespan, probably through prevention of processes such as redox imbalance, inflammation, obesity, diabetes, and atherosclerosis [21,22,23,24]. In skeletal muscles, resveratrol was shown to reduce injury of gastrocnemius in rats [25]. In other investigations, muscle regeneration was potentiated as a result of treatment with resveratrol in mice [26,27,28]. In rats, treatment with resveratrol was also shown to attenuate muscle wasting during mechanical unloading in a slow-twitch muscle type, the soleus [29,30]. Whether in mice resveratrol may favor muscle function and biology following disuse in a fast-twitch muscle type such as the gastrocnemius remains to be answered. 

Thus, we hypothesized that resveratrol may further attenuate proteolysis signaling, proteolytic events, atrophy signaling pathways, and structural phenotypic alterations and may improve muscle function in hindlimb muscles of mice exposed to seven days of unloading. Accordingly, in the gastrocnemius of mice exposed to a seven-day phase of hindlimb unloading with and without treatment with resveratrol, several molecular events involved in muscle mass maintenance the study objectives were to explore (1) markers of proteolysis, including systemic levels of troponin-I; (2) apoptosis; (3) atrophy signaling pathways (gene and protein expression); and (4) muscle phenotype and function. The model used in the present investigation was formerly well-validated in previous studies [15,16,17,18,28,31,32].

## 2. Methods

### 2.1. Animal Experiments

Female C57BL/6J mice (10 weeks old, weight ~20 g) were obtained from Harlan Interfauna Ibérica SL (Barcelona, Spain). Female mice were used for practical reasons, as previous investigations in our group had also been conducted on this type of animal [15,17,18]. Mice were kept under pathogen-free conditions in the animal house facility at Barcelona Biomedical Research Park (PRBB), with a 12:12 h light/dark cycle. 

**Figure 1:**
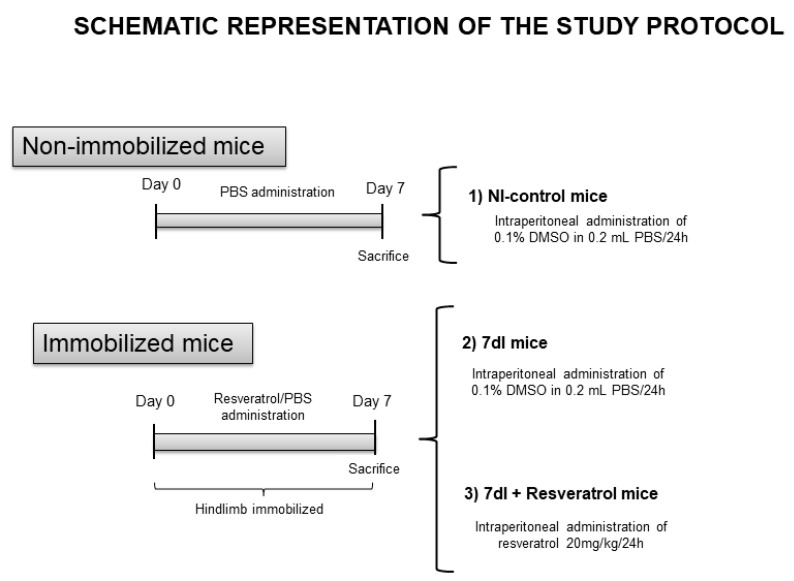
Graphical timeline representation of all the groups and the treatments administered to the mice in the study. Definition of abbreviations: PBS, phosphate-buffered saline; DMSO, dimethyl sulfoxide; mL, milliliter; mg, milligram; kg, kilogram; h, hour; I, immobilization.

The entire study protocol is shown in Figure 1. Mice were exposed to unilateral hindlimb unloading, as formerly reported, to replicate a model of disuse muscle atrophy [15,17,18]. The foot was maintained in a plantar-flexed position to induce the maximal atrophy of the target limb muscle. The mouse hindlimb was introduced into a 1.5 mL microcentrifuge tube (cast to immobilize the extremity) with cover and bottom lids detached [15,16,17,18,28,31,32]. Because the weight of the tube was approximately 0.6 g, it did not interfere with the usual mobility of the mice. Resveratrol (99.6% purity, C14H12O3, obtained from Polygonum cuspidatum sieb.et Zucc) was purchased from Fagron (Waregem, Belgium) and was prepared according to the specific manufacturer’s instructions under sterile conditions in our laboratory. Resveratrol was diluted in 0.1% dimethyl sulfoxide (10% DMSO concentration) as precisely indicated by the manufacturer and previous studies [28,33,34,35].

The following groups of animals were studied (n = 10/group, Figure 1): (1) Non-immobilized control mice (NI-control, vehicle 0.1% DMSO and normal saline solution, 0.2 mL intraperitoneal administration) from day 0 to day 7, (2) 7-days immobilized mice (7dI, left hindlimb immobilized for seven consecutive days, vehicle 0.1% DMSO and normal saline solution, 0.2 mL intraperitoneal administration) from day 0 to day 7, and (3) 7dI mice treated with resveratrol (7dI + Resveratrol, intraperitoneal administration, 20 mg/kg weight/24 h, suspended in 0.1% DMSO and 0.2 mL normal saline solution) [28,35,36,37] from day 0 to day 7. The rationale to administer resveratrol intraperitoneally was to ensure that each animal received exactly the same dose of the drug daily. Administration of resveratrol using other routes (water administration, during food, or oral) would not allow us to ensure an identical dose for each mouse. Furthermore, intraperitoneal administration avoids the absorption through the gastrointestinal tract and the first barrier of the hepatic metabolism, as generally happens in oral administration [38,39]. Accordingly, to ensure the optimal absorption of the resveratrol considering its short bioavailability in plasma, 30–60 min estimated in mice [40], intraperitoneal injection was the selected route due to the fact that it enters into the circulation faster than other routes (oral gavage). The three groups of mice (including the non-treated controls) were injected intraperitoneally, in order to control for a potential injection-induced stress response.

### 2.2. Ethics

All animal experiments were conducted in the animal facilities at PRBB. This was a controlled study designed in accordance with the ethical regulations on animal experimentation of the European Community Directive 2010/63/EU, the Spanish Legislation (*Real Decreto* 53/2013, BOE 34/11370–11421), and the European Convention for the Protection of Vertebrate Animals Used for Experimental and Other Scientific Purposes (1986). All animal experiments were approved by the Animal Research Committee at PRBB. Ethical approval was obtained by the Animal Research Committee (Animal Welfare Department in Catalonia, Spain, EBP-13-1485).

### 2.3. In Vivo Measurements in the Mice

Food and water were supplied ad libitum in the study period. In all the study animals, body weight and food intake were measured at every timepoint. Limb strength was determined on day 0 and right at the end of the unloading timepoints using a grip strength meter (Bioseb, Vitrolles Cedex, France), following previous studies [15,17,41,42,43] in which grip strength was also the endpoint parameter in the different experimental models. Grip strength was assessed in the four limbs at the same time in all the mice. Limb strength gain was calculated as the percentage of the measurements performed at the end of the study period with respect to the same measurements obtained at baseline (grip strength at the end of the study period–grip strength on day 0)/grip strength on day 0 × 100) in all the mice [15,17,44]. 

### 2.4. Sacrifice and Sample Collection

Mice from all the experimental groups were sacrificed after the corresponding unloading period (7 days). Anesthesia with sodium pentobarbital (0.1 mL, 60 mg/Kg body weight) was used prior to the sacrifice of the mice in order to ensure no suffering. The pedal and blink reflexes were assessed to check anesthetic depth. Gastrocnemius muscles were obtained from all the animals at the time of sacrifice and then were snap-frozen in liquid nitrogen to be thereafter stored at −80 °C until further use. Furthermore, for histological analyses, two additional fragments of the gastrocnemius muscles were fixed in 4% paraformaldehyde solution and were then embedded in optimum cutting temperature (OCT) for fiber-type analyses and morphometry and in paraffin for the apoptosis assay (See specific details below). 

### 2.5. Biological Analyses

#### 2.5.1. Muscle Fiber Type and Morphometry

On 10 µm frozen sections from gastrocnemius muscle of all study groups, immunofluorescence procedures with anti-MyHC I (ab11083, Abcam, Cambridge, UK) and anti-MyHC II (ab51263, Abcam) antibodies, respectively, were used to identify slow- and fast-twitch muscle fibers. In each muscle cross-section at least 100 fibers were measured and counted separately from all study groups of mice. Additionally, the hybrid fibers were counted separately from all study groups of animals.

#### 2.5.2. Terminal Deoxynucleotidyl Transferase-Mediated Uridine 5′-Triphosphate (UTP) Nick-End Labeling (TUNEL) Assay

In gastrocnemius muscle paraffin-embedded sections of all study groups of mice, the number of apoptotic nuclei were identified using the TUNEL assay (ApopTag^®^ Peroxidase In Situ Apoptosis Detection Kit, Merck-Millipore, Darmstadt, Germany). The manufacturer’s instructions and previously published studies were followed [42,43,45]. On this basis, in each muscle preparation, altered fibers were expressed as the percentage of the TUNEL-positive nuclei from the total number of counted nuclei following previously published methodologies [41]. A minimum number of 300 nuclei were counted in each muscle preparation. Final results corresponded to the mean values of the counts provided by the two trained independent observers (correlation coefficient 95%). 

#### 2.5.3. Ribonucleic Acid (RNA) Extraction

Total RNA was first isolated from the gastrocnemius muscle of mice using Trizol reagent, following the manufacturer’s protocol (Life Technologies, Carlsbad, CA, USA). Total RNA concentrations were determined spectrophotometrically using the NanoDrop 1000 (Thermo Scientific, Waltham, MA, USA).

#### 2.5.4. Procedures of Messenger (mRNA) Reverse Transcription (RT)

A single RT was performed from which all the target genes of the study were analyzed. First-stranded complementary deoxyribonucleic acid (cDNA) was generated from mRNA using oligo(dT)_12–18_ primers and Super-Script III reverse transcriptase, following the manufacturer’s instructions (Life Technologies).

#### 2.5.5. Quantitative Real Time-*Polymerase Chain Reaction* Amplification (qRT-PCR)

TaqMan-based qPCR reactions were performed using the ABI PRISM 7900HT Sequence Detector System (Life Technologies, Carlsbad, CA, USA) together with commercially available gene expression assays. The probes corresponding to the following genes involved in signaling of muscle atrophy were tested: ubiquitin-ligase atrogin-1(*Atrogin-1,* Mm00499523_m1, Life Technologies), ubiquitin-ligase muscle ring finger (MuRF)-1 (*Murf-1,* Mm01185221_m1, Life Technologies), FoxO1 (*Foxo1*, Mm00490671_m1, Life Technologies), and FoxO3 (*Foxo3,* Mm01185722_m1, Life Technologies). The housekeeping gene glyceraldehyde-3-phosphate dehydrogenase (*Gapdh*, Mm99999915_g1, Life Technologies) served as the endogenous control for the mRNA gene [46,47]. Reactions were run in duplicates, and mRNA data were collected and subsequently analyzed using the sequence detection system relative quantification software version 2.4 (Applied BioSystems, Waltham, MA, USA), in which the comparative C_T_ method (2^−ΔΔCT^) for relative quantification was employed [48].

#### 2.5.6. Immunoblotting of 1D Electrophoresis

Protein levels of the different molecular markers analyzed in the study were explored by means of immunoblotting procedures, as previously described [15,17,43]. 

The entire procedures were always conducted at 4 °C. Protein levels in crude homogenates were spectrophotometrically determined with the Bradford method, using triplicates in each case and bovine serum albumin (BSA) as the standard (Bio-Rad protein reagent, Bio-Rad Inc., Hercules, CA, USA). The final protein concentration in each sample was calculated from at least two Bradford measurements that were almost identical. Equal amounts of total protein (ranging from 5 to 60 µg, depending on the antigen and antibody) from crude muscle homogenates were always loaded onto the gels, as well as identical sample volumes/lanes. For the purpose of comparisons among the different groups of experimental and control rodents, muscle sample specimens were always run together and kept in the same order. Three fresh 10-well mini-gels were always simultaneously loaded for each of the antigens. Experiments were confirmed at least twice for all the antigens analyzed in the investigation. Fresh gels were specifically loaded for each of the antigens in muscle specimens of all mice in most of cases. However, in a few cases, antigens were identified from stripped membranes (see below). 

Proteins were then separated by electrophoresis, transferred to polyvinylidene difluoride (PVDF) membranes, blocked with BSA, and incubated overnight with selective primary antibodies. Levels of total acetylated proteins, signaling pathways, and downstream targets were identified in the gastrocnemius using specific primary antibodies: total acetylated proteins (anti-acetyl-lysine antibody, Santa Cruz Biotechnology, Santa Cruz, CA, USA), FoxO1 (anti-FoxO1 antibody, Merck-Millipore, Darmstadt, Germany), FoxO3 (anti-FoxO3 antibody, Origene, Herford, Germany), total ubiquitinated proteins (anti-ubiquitinated proteins antibody, Boston Biochem, Cambridge, MA, USA), 20S proteasome subunit C8 (anti-C8 antibody, Biomol, Plymouth Meeting, PA, USA), atrogin-1 (anti-atrogin-1 antibody, Acris), MuRF-1 (anti-MURF-1 antibody, Santa Cruz Biotechnology), and GAPDH (anti-GAPDH antibody, Santa Cruz Biotechnology). Antigens from all samples were detected with horseradish peroxidase (HRP)-conjugated secondary antibodies and a chemiluminescence kit. For each of the antigens, samples from the different groups were always detected in the same picture under identical exposure times.

Acetylation levels of the transcription factors FoxO1 and FoxO3 were detected as previously reported [15,17]. Acetylated levels of the target markers were calculated as the ratio of the acetylated-to-total protein content for each of the markers, as previously described [15,17].

PVDF membranes were scanned with the Molecular Imager Chemidoc XRS System (Bio-Rad Laboratories, Hercules, CA, USA) using the software Quantity One version 4.6.5 (Bio-Rad Laboratories). Optical densities of specific proteins were quantified using the software Image Lab version 2.0.1 (Bio-Rad Laboratories). Final optical densities obtained in each specific group of mice corresponded to the mean values of the different samples (lanes) of each of the study antigens. To validate equal protein loading among various lanes, the glycolytic enzyme GAPDH was used as the protein loading control in all the immunoblots. The target protein in each immunoblot was indicated by an arrow in the representative images, included below. Negative control experiments enabled the identification of the target proteins as well as the positive control in the case of FoxO3 protein. Moreover, the measured ubiquitinated proteins are also indicated by an arrow. 

#### 2.5.7. Protein Catabolism

Protein degradation was explored on the basis of the rate of production of free tyrosine from tissue proteins, as previously described [17,49,50,51]. As muscles cannot synthesize or degrade this amino acid, its accumulation reflects the net degradation of proteins. The results are expressed as nmol of tyrosine/mg of muscle/2 h of incubation. 

#### 2.5.8. Enzyme-Linked Immunosorbent Assay (ELISA) Plasma Skeletal Muscle Troponin-I Levels

Skeletal muscle troponin-I levels were quantified in plasma of all study groups of mice: NI-control, 7dI, 7dI + Resveratrol, using a specific sandwich ELISA kit (Life Diagnostics Inc., West Chester, PA, USA), as previously described [17,52,53,54,55]. Intra-assay coefficients of variation for the plasma skeletal muscle troponin-I levels ranged from 2% to 10%. Because all the samples were analyzed on the same day, no inter-assay coefficients of variation could be calculated. 

### 2.6. Statistical Analysis

All the statistical analyses were performed using the Statistical Package for the Social Sciences (Portable SPSS, PASW statistics 18.0 version for Windows, SPSS Inc., Chicago, IL, USA). The results are presented as mean values (standard deviation). Normality of the study variables was examined using Shapiro–Wilk test. Results of the variables food intake and percentage of change of total body weight, limb strength, and muscle structure are represented in Table 1 and Table 2. The biological variables are represented in Figures. Potential differences among the study groups were explored using one-way analysis of variance (ANOVA) and Tukey’s post hoc analysis to adjust for multiple comparisons among the study groups. Two levels of comparisons were established for all the study variables: (1) comparisons between the non-immobilized control group and the 7dI non-treated mice and (2) comparisons between the 7dI + Resveratrol mice and the 7dI non-treated rodents. The results are described on the basis of these two types of comparisons. A level of significance of *p* ≤ 0.05 was established.

## 3. Results

### 3.1. Physiological Characteristics of the Study Animals

#### 3.1.1. Non-Immobilized versus Unloading Conditions

Compared with non-immobilized (NI)-control animals, in unloaded mice, total body and gastrocnemius weight and limb strength gain were significantly reduced, while food intake was not modified (Table 1). 

#### 3.1.2. Unloading with Resveratrol versus Unloading

No significant differences were seen in food intake, total body, and gastrocnemius weight between 7 days immobilized (7dI) + Resveratrol mice and the non-treated unloaded mice (Table 1). Limb strength gain did not further decrease in the immobilized mice treated with resveratrol (Table 1). 

### 3.2. Structural Phenotypic Characteristics

#### 3.2.1. Non-Immobilized versus Unloading Conditions

**Figure 2:**
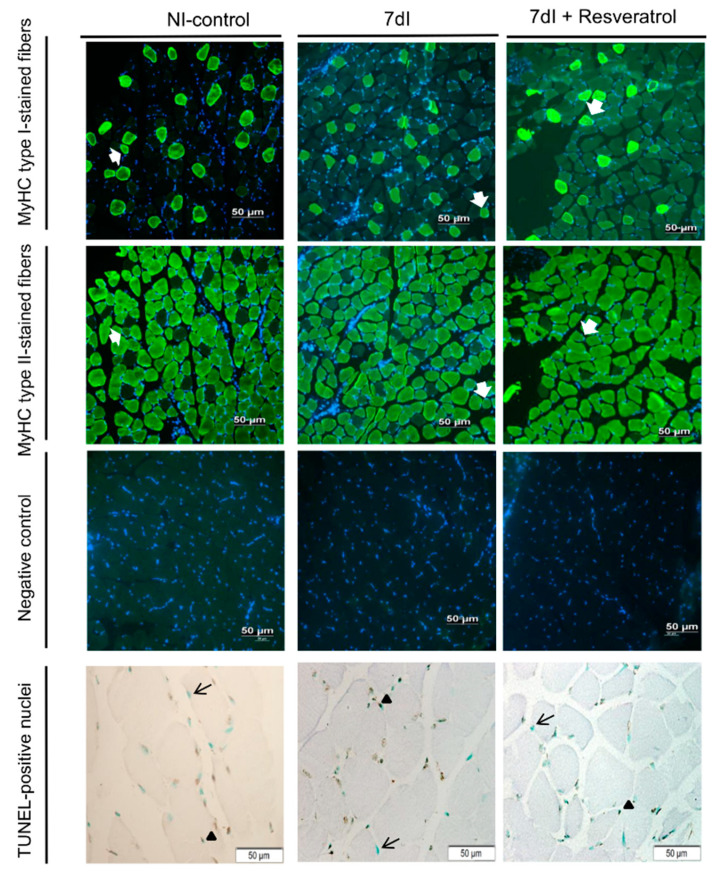
Representative examples of the gastrocnemius muscle in animals of the all study groups of mice. Myofibers are stained in green in the top and middle panels, type I in the top panel and type II in the middle-up panel, and negative controls with no staining (middle-down panels). Hybrid fibers (white arrows) are seen in both up panels. Myofibers of positively stained nuclei (brown color, black arrowheads) and negatively stained nuclei (no staining, black arrows) for the TUNEL assay are seen in the bottom panel. Definition of abbreviations: MyHC, myosin heavy chain; TUNEL, terminal deoxynucleotidyl transferase-mediated uridine 5′-triphosphate nick-end labeling; NI, non-immobilized; I, immobilization.

Compared with NI-control animals, in the gastrocnemius of unloaded mice, CSA of both type I and type II muscle fibers significantly decreased, while the proportions of hybrid fibers increased (Table 2 and Figure 2). The number of TUNEL-positive nuclei was significantly greater in the unloaded animals than in the non-immobilized controls (Table 2 and Figure 2). Fiber type proportions of both slow- and fast-twitch and hybrid fibers did not significantly differ between groups (Table 2 and Figure 2).

#### 3.2.2. Unloading with Resveratrol versus Unloading

No significant differences were observed in fiber type proportions between non-treated unloaded and the 7dI + Resveratrol mice, while CSA of both type I and type II muscle fibers significantly increased in the gastrocnemius of the latter group (Table 2 and Figure 2). Fiber type proportions and CSA of hybrid fibers did not significantly differ between treated and non-treated immobilized rodents (Table 2 and Figure 2). Number of TUNEL-positive nuclei was significantly lower in the unloaded treated animals (Table 2 and Figure 2).

### 3.3. Muscle Proteolysis

#### 3.3.1. Non-Immobilized versus Unloading Conditions

**Figure 3:**
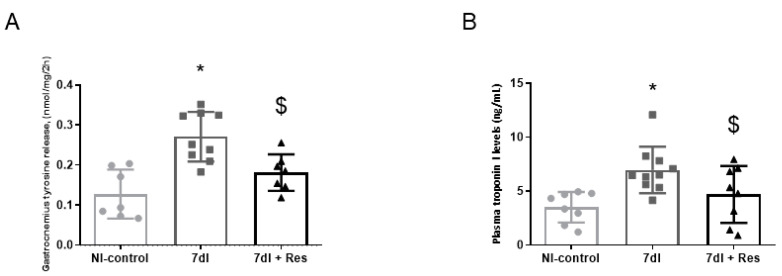
(**A**) Mean values and standard deviations of the variable tyrosine release (nmol/mg/2 h) of the gastrocnemius muscle of the different study groups of mice. (**B**) Mean values and standard deviations of the variable plasma troponin-I (ng/mL) of the gastrocnemius muscle of the different study groups of mice. Statistical significance is represented as follows: * *p* < 0.05 between 7dI animals and the non-immobilized mice; $ *p* < 0.05 the group of resveratrol-treated mice compared with the 7dI group. Definition of abbreviations: NI, non-immobilized; I, immobilization; nmol, nanomol; mg, milligram; h, hour; ng, nanogram; mL, mililiter.

In the gastrocnemius of unloaded animals, levels of both tyrosine release and plasma troponin I levels were significantly greater than in the non-immobilized controls (Figure 3A,B, respectively).

#### 3.3.2. Unloading with Resveratrol versus Unloading

In gastrocnemius of 7dI + Resveratrol mice, both tyrosine release and plasma troponin I levels were significantly lower than those detected in non-treated unloaded mice (Figure 3A,B, respectively).

### 3.4. Markers of Proteolysis

#### 3.4.1. Non-Immobilized versus Unloading Conditions

**Figure 4:**
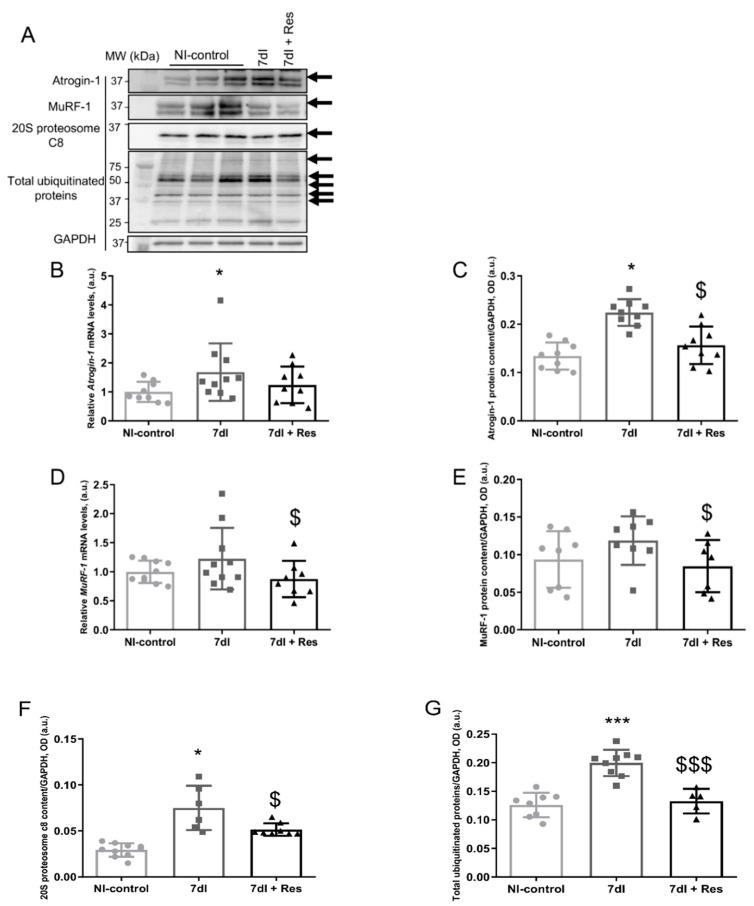
(**A**) Representative immunoblots of atrogin-1, MuRF-1, 20 S proteasome alpha subunit C8, total ubiquitinated proteins, and GAPDH proteins in the gastrocnemius muscle of all study groups of mice. The analyzed bands are indicated with specific arrows. (**B**) Mean values and standard deviations of gene expression of *Atrogin-1* in the gastrocnemius muscle of the different study groups of mice. (**C**) Mean values and standard deviations of atrogin-1 protein content in the gastrocnemius muscle of the different study groups of mice, as measured by optical densities in arbitrary units (OD, a.u.). (**D**) Mean values and standard deviations of gene expression of *MuRF-1* in the gastrocnemius muscle of the different study groups of mice. (**E**) Mean values and standard deviations of MuRF-1 protein content in the gastrocnemius muscle of the different study groups of mice, as measured by optical densities in arbitrary units (OD, a.u.). (**F**) Mean values and standard deviations of C8-20S protein content in the gastrocnemius muscle of the different study groups of mice, as measured by optical densities in arbitrary units (OD, a.u.). (**G**) Mean values and standard deviations of total ubiquitinated proteins content in the gastrocnemius muscle of the different study groups of mice, as measured by optical densities in arbitrary units (OD, a.u.). Statistical significance is represented as follows: * *p* < 0.05 between 7dI animals and the non-immobilized mice, $ *p* < 0.05 the group of resveratrol-treated mice compared with the 7dI group, *** *p* < 0.001 between 7dI animals and the non-immobilized mice, $$$ *p* < 0.001 the group of resveratrol-treated mice compared with the 7dI group. Definition of abbreviations: MuRF-1, muscle RING-finger protein-1; GAPDH, glyceraldehyde-3-phosphate dehydrogenase; MW, molecular weight; kDa, kilodalton; NI, non-immobilized; I, immobilization; mRNA, messenger ribonucleic acid; a.u., arbitrary units; OD, optical densities.

Compared with non-immobilized animals, in the gastrocnemius of unloaded mice, ubiquitin-ligase atrogin-1 (atrogin-1, gene and protein expression), proteasome content, and total protein ubiquitination levels were significantly higher, while levels of muscle ring finger (MuRF)-1 (gene and protein expression) did not significantly differ (Figure 4A–G, respectively). 

#### 3.4.2. Unloading with Resveratrol versus Unloading

In gastrocnemius of 7dI + Resveratrol mice, atrogin-1 protein content, MuRF-1 (gene and protein expression), proteasome content, and total protein ubiquitination levels were lower than in unloaded mice, whereas atrogin-1 gene expression did not significantly differ (Figure 4A–G, respectively).

### 3.5. Muscle Atrophy Signaling Markers

#### 3.5.1. Non-Immobilized versus Unloading Conditions

**Figure 5:**
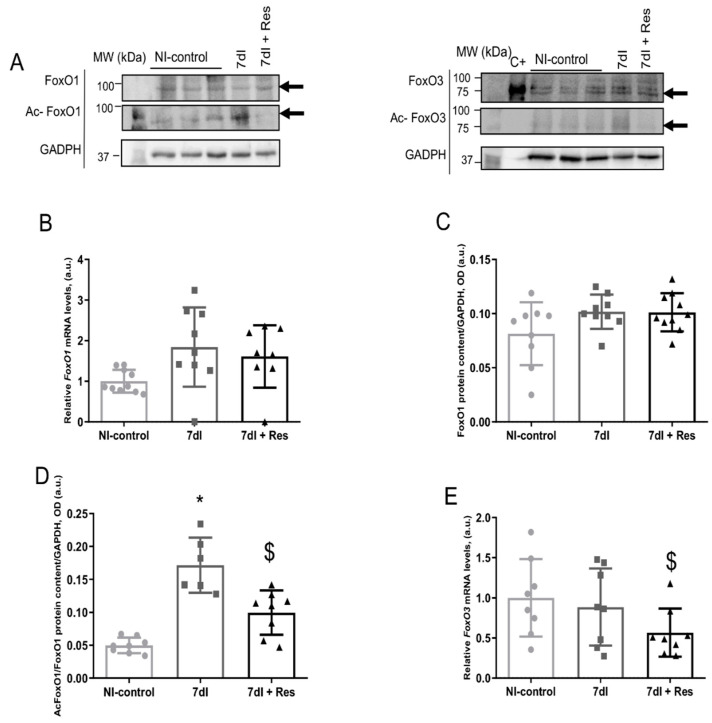

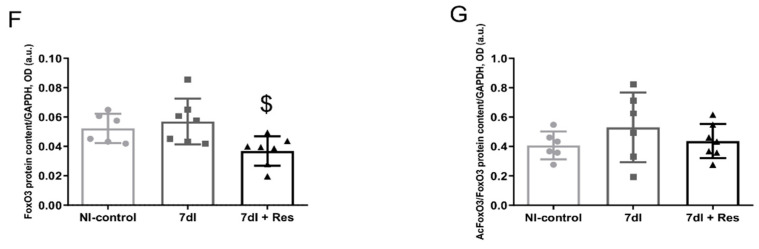
(**A**) Representative immunoblots of FoxO1, acetylated FoxO1, FoxO3, acetylated FoxO3, and GAPDH proteins in the gastrocnemius muscle of all study groups of mice. The analyzed bands are indicated with specific arrows. (**B**) Mean values and standard deviations of gene expression of *FoxO1* in the gastrocnemius muscle of the different study groups of mice. No statistical differences were observed between groups. (**C**) Mean values and standard deviations of FoxO1 protein content in the gastrocnemius muscle of the different study groups of mice, as measured by optical densities in arbitrary units (OD, a.u.). No statistical differences were observed between groups. (**D**) Mean values and standard deviations of acetylated FoxO1 protein content in the gastrocnemius muscle of the different study groups of mice, as measured by optical densities in arbitrary units (OD, a.u.). (**E**) Mean values and standard deviations of gene expression of *FoxO3* in the gastrocnemius muscle of the different study groups of mice. (**F**) Mean values and standard deviations of FoxO3 protein content in the gastrocnemius muscle of the different study groups of mice, as measured by optical densities in arbitrary units (OD, a.u.). (**G**) Mean values and standard deviations of acetylated FoxO3 protein content in the gastrocnemius muscle of the different study groups of mice, as measured by optical densities in arbitrary units (OD, a.u.). No statistical differences were observed between groups. Statistical significance is represented as follows: * *p* < 0.05 between 7dI animals and the non-immobilized mice; $ *p* < 0.05 the group of resveratrol-treated mice compared with the 7dI group. Definition of abbreviations: FoxO1, transcription factor fork-head box O1; FoxO3, transcription factor fork-head box O3; ac-, acetylated; *p*-, phosphorylated; GAPDH, glyceraldehyde-3-phosphate dehydrogenase; MW, molecular weight; kDa, kilodalton; NI, non-immobilized; I, immobilization; mRNA, messenger ribonucleic acid; a.u., arbitrary units; OD, optical densities.

Both gene expression and protein levels of FoxO1 and FoxO3 (acetylated levels included) did not significantly differ between the unloaded and the control mice. Acetylated levels of FoxO1 were significantly higher in the former animals than in the latter group (Figure 5A–G, respectively).

#### 3.5.2. Unloading with Resveratrol versus Unloading

In the limb muscle of 7dI + Resveratrol mice compared with the controls, gene expression and protein levels of FoxO1 did not significantly differ, acetylated FoxO1 decreased, both FoxO3 gene expression and protein content diminished, and no significant differences between the two groups were observed in acetylated FoxO3 levels (Figure 5A–G, respectively).

## 4. Discussion

Atrophy of the muscle fibers as a result of prolonged bed rest is common in clinical settings of patients with severe illness, acute exacerbations of chronic respiratory and cardiac conditions, cancer, surgery, and trauma. Following hospital discharge, patients usually experience loss of muscle mass, especially of the lower limbs. This has a negative impact on the patients’ performance and quality of life, which further aggravates the underlying sarcopenia/cachexia that the patients may have in the context of chronic disease. In the unloading control muscles, treatment of the animals with resveratrol elicited several modifications, such as a significant decline in the number of TUNEL-positive nuclei, tyrosine release, plasma troponin I, atrogin-1 content, total protein ubiquitination levels, acetylated FoxO1 levels, and total FoxO3 levels. Furthermore, the size of both slow- and fast-twitch muscle fibers also improved in the gastrocnemius of the unloaded mice with resveratrol.

Resveratrol, which is a natural polyphenol that is obtained from peanuts and grapes and other plants, exerts important effects within cells through targeting several processes such as inflammation, oxidative stress, and atherosclerosis and was shown to increase the lifespan of certain species [22,23,24,56]. Resveratrol was also shown to favor the physiological adaptation and energy content in patients with peripheral muscle fatigue during aerobic exercise [57]. Muscle mass and function also significantly improved in elderly patients in response to combined aerobic exercise training and resveratrol treatment [58]. Additionally, resveratrol may also exert antioxidant effects that alter the production of oxygen free radicals induced by exercise [59].

Despite that the measurement of gastrocnemius muscle strength using rather invasive procedures (in vitro contractility studies or measurements of isometric torque) would have been more precise and specific, a non-invasive approach was chosen in the current investigation. As such, limb muscle strength of the four limbs was used to assess and monitor longitudinally the variations in this physiological parameter observed in all the groups of mice throughout the study period. The non-invasive methodologies allowed the monitoring of muscle strength throughout the study protocol as well as the quantification of this parameter in the mice at different timepoints. Treatment with resveratrol prevented a further decline in limb muscle strength in the immobilized treated animals.

In the current investigation, resveratrol induced several beneficial therapeutic effects in the gastrocnemius muscle of the mice exposed to a seven-day period of unloading. For instance, a decrease in the expression levels of several biological mechanisms involved in muscle proteolysis and apoptosis, including signaling, was observed in the unloaded mice treated with the polyphenolic compound. Nonetheless, muscle mass, body weight, or limb strength did not improve beyond levels seen in the controls in response to resveratrol therapy during unloading. It is likely that a longer duration of the treatment with resveratrol might have induced functional and/or structural benefits in the gastrocnemius muscle, as shown in previous experiments in mice [60].

Protein levels of atrogin-1 and those of the 20S proteasome C8 subunit and protein ubiquitination significantly decreased in the limb muscle of the unloaded animals treated with resveratrol, implying that reduced muscle proteolysis was probably mediated by this ligase. These observations suggest that resveratrol also induced beneficial effects on proteolysis in the immobilized muscle. Levels of MuRF-1, however, did not experience any significant modification among the study groups of mice. Another relevant finding was the significant reduction seen in TUNEL-positive nuclei in the gastrocnemius muscle of the unloaded mice treated with resveratrol. These findings suggest that resveratrol attenuated nuclear apoptosis during disuse muscle atrophy. These results also imply that the polyphenolic compound contributes to the physiological adaptations to the process that deconditions skeletal muscle fibers [60,61].

In previous studies [26,28], treatment of unloaded mice also favored muscle regeneration in rodents. Additionally, muscle injury (contusion model) was also attenuated as a result of treatment with resveratrol [27]. In aged rats exposed to tail suspension, muscle mass recovery and larger fast-twitch fibers were also observed in response to treatment with resveratrol [26]. Furthermore, in type I diabetic mice, mitochondrial membrane potential was restored within the myofibers of the target muscles [62]. Whether additional beneficial effects of treatment with resveratrol can be identified in skeletal muscles of animals exposed to longer periods of time or in combination with recovery should be explored in future investigations.

In the gastrocnemius of the immobilized mice, FoxO3 protein levels did not significantly differ from those observed in the control animals. Nonetheless, a decline in FoxO3 protein levels was detected in the limb muscle of the immobilized mice treated with resveratrol. These are relevant findings that suggest that FoxO3 transcription factor may be a player in atrophy signaling during immobilization [33,63] and that resveratrol contributes to attenuating FoxO3 activity, as has been also shown to occur in other models [63]. Importantly, acetylated levels of FoxO1 also decreased in the gastrocnemius muscle in response to treatment with resveratrol during inactivity. Similar observations were reported in cardiomyocytes exposed to hypoxia, in which resveratrol treatment attenuated apoptosis levels via FoxO1 activity [33].

### 4.1. Study Limitations

A potential limitation was the lack of a significant improvement in limb muscle strength observed in the immobilized mice treated with resveratrol. These observations need to be taken cautiously, as resveratrol rather prevented a further decline in muscle strength in mice exposed to a 7-day period of hindlimb immobilization. Another limitation is related to the doses used in the present study compared with other investigations. This may lead to discrepant results among studies. In fact, the different conformations of resveratrol (cis- and trans-) [64], experimental models of in vivo and in vitro design, and the duration of the experimental protocols may account for the discrepancies observed among investigations [28,29,30,35,64].

Another issue that deserves attention is the route of resveratrol administration employed in the current study. Other routes may have yield different results [38,39,40] in animal models, and in the case of studies conducted on patients, the oral administration should be preferred or local inoculation through nanoparticles [65,66].

### 4.2. Conclusions

Resveratrol treatment of mice exposed to a seven-day period of unloading prevented body and muscle weight and limb strength loss, while an improvement in muscle proteolysis, proteolytic markers, atrophy signaling pathways, apoptosis, and muscle fiber CSA was observed in the gastrocnemius muscle. These findings may have potential therapeutic implications in the management of disuse muscle atrophy in clinical settings.

## Figures and Tables

**Table 1:** Physiological parameters in all experimental groups of mice.

	NI-Control	7DI	7DI + Resveratrol
Food intake (g/24 h)	3.20 (0.10)	3.24 (0.18)	3.25 (0.078)
Total body weight gain (%)	+6.25 (1.02)	−0.78 (3.46) **	+1.03 (1.87)
Gastrocnemius weight (g)	0.113 (0.009)	0.097 (0.011) *	0.099 (0.011)
Limb strength gain (%)	+13.44 (6.10)	−14.22 (8.97) *	−9.04 (15.21)

Variables are presented as mean (standard deviation). Definition of abbreviations: g, gram; h, hour; NI, non-immobilized; I, immobilization. Statistical significance is represented as follows: ** *p* < 0.01; * *p* < 0.05 between 7dI animals and the non-immobilized mice.

**Table 2:** Structural characteristics of the gastrocnemius in all animal groups.

	NI-Control	7DI	7DI + Resveratrol
Muscle fiber type, %			
Type I fibers	15.40 (1.99)	16.88 (2.70)	14.60 (2.35)
Type II fibers	84.60 (5.74)	83.12 (2.70)	85.40 (2.35)
Cross-sectional area, µm^2^			
Type I fibers	1240.46 (122.93)	882.28 (199.96) ***	1032.89 (160.48) ^$^
Type II fibers	1252.52 (95.32)	879.85 (183.05) **	1065.02 (133.06) ^$^
Muscle hybrid fiber, %	0.59 (0.10)	3.55 (1.61) **	2.37 (2.45)
Cross-sectional area hybrid fibers, µm^2^	1012.90 (57.85)	881.97 (270.88)	855.71 (238.68)
Number of apoptotic nuclei (TUNEL), %	52.53 (9.16)	69.80 (5.58) *	56.98 (7.14) ^$^

Variables are presented as mean (standard deviation). Definition of abbreviations: NI, non-immobilized; I, immobilization; µm, micrometer; TUNEL, terminal deoxynucleotidyl transferase-mediated uridine 5′-triphosphate nick-end labeling. Statistical significance is represented as follows: *** *p* < 0.001; ** *p* < 0.01; * *p* < 0.05 between 7dI animals and the non-immobilized mice; ^$^ *p* < 0.05 the group of resveratrol-treated mice compared with the 7dI group.

## Data Availability

The data sets used and/or analyzed during the current study are available from the corresponding author on reasonable request.
